# Protein Quantification by Derivatization-Free High-Performance Liquid Chromatography of Aromatic Amino Acids

**DOI:** 10.1155/2016/7374316

**Published:** 2016-07-31

**Authors:** Almut Hesse, Michael G. Weller

**Affiliations:** Division 1.5 Protein Analysis, Bundesanstalt für Materialforschung und -prüfung (BAM), Richard-Willstätter-Strasse 11, 12489 Berlin, Germany

## Abstract

Amino acid analysis is considered to be the gold standard for quantitative peptide and protein analysis. Here, we would like to propose a simple HPLC/UV method based on a reversed-phase separation of the aromatic amino acids tyrosine (Tyr), phenylalanine (Phe), and optionally tryptophan (Trp) without any derivatization. The hydrolysis of the proteins and peptides was performed by an accelerated microwave technique, which needs only 30 minutes. Two internal standard compounds, homotyrosine (HTyr) and 4-fluorophenylalanine (FPhe) were used for calibration. The limit of detection (LOD) was estimated to be 0.05 *µ*M (~10 *µ*g/L) for tyrosine and phenylalanine at 215 nm. The LOD for a protein determination was calculated to be below 16 mg/L (~300 ng BSA absolute). Aromatic amino acid analysis (AAAA) offers excellent accuracy and a precision of about 5% relative standard deviation, including the hydrolysis step. The method was validated with certified reference materials (CRM) of amino acids and of a pure protein (bovine serum albumin, BSA). AAAA can be used for the quantification of aromatic amino acids, isolated peptides or proteins, complex peptide or protein samples, such as serum or milk powder, and peptides or proteins immobilized on solid supports.

## 1. Introduction

Amino acid analysis (AAA) seems to lose some importance over the recent years. One of the reasons might be the fact that AAA needs sophisticated analytical devices and remained a complex, relatively expensive, and nontrivial task. Conventional AAA can be performed based on precolumn derivatization, for example, with phenylisothiocyanate [1], FMOC (fluorenylmethyloxycarbonyl chloride) [2, 3], OPA (*o*-phthaldialdehyde) [4], or 6-aminoquinolyl-N-hydroxysuccinimidyl-carbamate [5]. An important advantage of this approach is the use of reversed-phase (RP) chromatography. However, a very good separation performance is required to avoid peak overlap. Another traditional option is postcolumn derivatization, which needs ion-exchange columns run with highly optimized buffers and dedicated postcolumn derivatization devices. The most important chemistry is based on ninhydrin [6], leading to highly colored products, which can be detected photometrically in the visible range. Moore et al. also published the development of the first automated amino acid analyzer [7, 8]. In addition, gas-chromatographic (GC) techniques have been applied for amino acid analysis for a long time [9]. This approach always requires one or several derivatization steps to make the analytes sufficiently volatile [10]. Recently, GC was applied for chiral amino acid analysis on spacecrafts [11], such as the robotic lander Philae [12]. A capillary-electrophoretic method based on laser-induced fluorescence [13] achieved limits of detection about 9 · 10^−21^ mol. More recently, mass-spectrometric methods for amino acid analysis emerged [14]. LC-MS/MS-based methods often do not need any derivatization [15]; however, they do not achieve complete chromatographic separation in many cases; they heavily rely on the mass-spectrometric selectivity. Also a more recent development is the use of ICP-MS [16–20] and hence the detection of the sulphur-containing amino acids cysteine and methionine [17]. Plenty of powerful methods for amino acid analysis are available today. Unfortunately, for a peptide or protein analysis, a prior hydrolysis step is required, which may need one day or more and often introduces major sources of error. Even a “total analysis” of amino acids lacks some of them; particularly cysteine and tryptophan are not included in the standard runs. Furthermore, glutamine and asparagine are converted to the respective acids during hydrolysis. Extra determination of cysteine, tryptophan, and other difficult amino acids would increase the cost and effort so much that this is nearly never performed. This means that “total amino acid analysis” has no closed mass balance and some assumptions about the sequence or amino acid composition have to be made.

Due to complexity, duration, cost, and effort, amino acid analysis is not used in many cases, in which it would be helpful. In biochemistry or food analysis, either colorimetry [21] or methods based on nitrogen determination are performed routinely. The vast majority of protein analyses in biochemistry are performed by colorimetric methods, such as the bicinchoninic acid assay (BCA assay [22]). Major problems are the different response factors of proteins and a large number of interferences. Therefore, these assays have to be regarded as semiquantitative. Another option for the quantification of proteins and peptides is based on the UV absorbance of proteins [23] caused by tryptophan and tyrosine at about 280 nm or of the peptide backbone in the range of 200–230 nm. In relatively pure and concentrated samples, the determination of the absorbance at 280 nm might be useful, if a suitable reference protein is available. In the lower UV range, the sensitivity is higher, but interference of solvents, buffers, and other additives is often prohibitive. In food analysis, the determination of nitrogen [24], for example, with the Kjeldahl method [25], prevails, which is relatively precise, but is based on toxic catalysts and can be easily manipulated, which became evident during the Chinese milk scandal. In this case, the nitrogen-rich synthetic compound melamine was used illegally to increase the apparent protein content [26].

**Figure 1:**
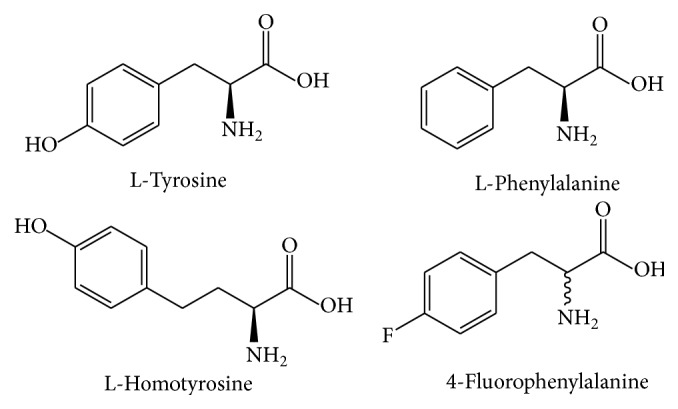
Aromatic amino acids L-tyrosine (Tyr, Y) and L-phenylalanine (Phe, F) and their respective internal standards, L-homotyrosine (HTyr) and 4-fluoro-DL-phenylalanine (FPhe).

In contrast to most amino acids, which either need to be chemically derivatized or separated on very specialized ion-exchange columns with a fully dedicated amino acid analyzer, the determination of aromatic amino acids can be achieved by standard reversed-phase chromatography (HPLC) with UV, fluorescence, or mass-spectrometric detection. Although this approach has been proposed some years ago [27], it was not explored in depth. The textbook of Molnar-Perl [28], which gives a very good overview to many techniques for amino acid analysis based on gas chromatography, high-performance liquid chromatography, and capillary electrophoresis, does not even mention this approach. For peptides and proteins, usually a hydrolysis with hydrochloric acid for about 24 hours is performed [29]. We finally changed to an accelerated method based on microwave digestion, which is completed in only 30 min. For the direct analysis of aromatic amino acids in serum of kidney disease patients by fluorescence detection, a paper was published in 2011 [30]. In addition, for the test for phenylketonuria, the direct analysis of aromatic amino acids was shown [31]. However, we would like to recommend this method not only for the determination of the respective hydrophobic amino acids, but also for the fast and precise quantification of suitable peptides, pure proteins, and even complex protein mixtures. As an abbreviation, we suggest the use of AAAA for* aromatic amino acid analysis*. Tryptophan was excluded in most of our work due to its lack of stability during acidic hydrolysis, but it could be perfectly quantified in the same chromatographic run, if no acidic hydrolysis is necessary. The conceptual advantages and disadvantages of aromatic amino acid analysis (AAAA) are the following: No derivatization and minimum sample preparation is required, the use of routine HPLC equipment is possible, calibration is straightforward and the measurements are very precise and might be even metrologically traceable, and in combination with a microwave digestion, a short analysis time can be achieved. Due to the combination of an aqueous sample with a reversed-phase separation, a significant clean-up effect is achieved, which leads to surprisingly little interference in complex samples, such as human serum, even with essentially non-selective UV detection at short wavelengths. In case of highest precision requirements, the concept might be extended to mass-spectrometric variants based on isotope dilution using ^13^C-labelled amino acids (not performed here). One could object that the determination of only one or two of more than twenty amino acids would hardly lead to correct protein quantification. If the sequence of the protein is known, which is assured for most proteins or peptides today, it is straightforward and accurate to calculate the protein mass from the phenylalanine or tyrosine content. Exactly the same approach is used in the case of sulphur-containing amino acids and their precise determination by elemental analysis (see above). Only in the case of complex and unknown mixtures of peptides and/or proteins, some uncertainty remains about the composition. Based on the long-term experience in food or clinical analysis, it can be assumed that conversion factors, which have to be determined experimentally or obtained from tables or textbooks, are quite stable and will lead to reliable protein determinations. To improve reproducibility of AAAA, we used two rare amino acid derivatives (Figure 1) as internal standards. Since UV detection should be used preferentially in this work, the common approach via isotopically labelled internal standards was not used here. The obvious disadvantage of chemically distinct derivatives is their potentially different behavior during hydrolysis and other handling steps; however we had no indications of systematic errors, yet.

## 2. Materials and Methods

### 2.1. Reagents

For the internal standard p-fluoro-DL-phenylalanine (CAS 51-65-0), Sigma-Aldrich Chemie GmbH (F5251), and L-homotyrosine hydrobromide (CAS 141899-12-9), IRIS Biotech GmbH (HAA6750.0001), were used. The calibration solution was based on a mix of 17 amino acids, NIST standard reference material (NIST 2389a): amino acids (2.50 ± 0.07 mM L-alanine, 2.51 ± 0.07 mM L-arginine, 2.50 ± 0.08 mM L-aspartic acid, 1.23 ± 0.06 mM L-cystine, 2.50 ± 0.08 mM L-glutamic acid, 2.52 ± 0.07 mM glycine, 2.52 ± 0.07 L-histidine, 2.44 ± 0.11 mM L-isoleucine, 2.44 ± 0.11 mM L-leucine, 2.41 ± 0.17 mM L-lysine, 2.51 ± 0.07 mM L-methionine, 2.55 ± 0.09 mM L-phenylalanine, 2.46 ± 0.11 mM L-proline, 2.44 ± 0.11 mM L-serine, 2.49 ± 0.07 mM L-threonine, 2.54 ± 0.08 mM L-tyrosine, and 2.51 ± 0.10 mm L-valine) in 0.1 N HCl. L-Tryptophan, L-asparagine, and L-glutamine have been taken from the L-amino acid set (Sigma-Aldrich LAA21). Furthermore, 6 M HCl (Thermo: Sequanal Grade Prod. number 24308), acetonitrile (Roth, LC/MS grade), and trifluoroacetic acid (TFA) from Fluka (LC-MS Ultra, eluent additive for UHPLC-MS, 99%) were used. For the examination of the method including hydrolysis, the NIST standard reference material SRM 927e, Bovine Serum Albumin, was used: a solution of BSA of about 7% (based on AAA): 67.38 g/L ± 1.38 g/L, BSA concentration (Biuret method): 69.58 g/L ± 1.30 g/L, density: 1.0182 ± 0.0002 g/mL, theoretical mass: 66398.1 g/mol, mass (decreasing order of abundance): 66431.3 ± 0.9, 66548.9 ± 0.8, 66458 ± 1, and 66590 ± 6. Human serum (Biochrom GmbH S01049.1-01), mouse serum (Kraeber GmbH H8090115-01 0368C), bovine IgG (Sigma 65009-5, CAS 9007 83-4), and dried milk powder (Saliter DE BY 7144 EF) were used as complex samples. Toyopearl AF-Tresyl-650M Bulk Media (Tosoh Bioscience LLC, Sigma-Aldrich 814471), Fractogel EMD Epoxy (M) (Merck 1.16961.0010), and Trisopor Amino 1500 (VitroBio GmbH) were used as solid affinity supports for immobilization of bovine serum albumin BSA (BSA, Sigma-Aldrich A7511—5 g, purity of 97%).

### 2.2. Materials

HPLC analyses were performed using an Agilent Technologies 1260 Infinity series system consisting of an 1260 Infinity Agilent Bio Quaternary pump G5611A, an 1260 Infinity Diode Array Detector (DAD) G4212B with an Max-Light Cartridge Cell 60 mm BIO G5615-60017, an 1260 Infinity HiP Bio ALS G5667A Automated Sample Injector, an 1290 Infinity Autosampler Thermostat G1330B, and a thermostatted column oven Compartment 1290 Infinity TCC G1316C. The system was controlled by Agilent ChemStation software. Chromatographic separation was performed on a reversed-phase column (Agilent Technologies AdvanceBio Peptide Map 2.1 × 150 mm, 2.7 *µ*m) with a precolumn (Agilent AdvanceBio Peptide Map Fast Guard 2.1 mm × 5 mm, 2.7 *µ*m). For the standard hydrolysis, a vacuum hydrolysis tube (1 mL; Thermo 29570) was used. For the microwave hydrolysis, a CEM Microwave Discover system with a CEM Protein Hydrolysis unit and a CEM vacuum pump was used. The samples were evaporated by a vacuum concentrator Christ RVC 2-18 CD plus with the vacuum pump Vacuubrand MZ 2C NT+AK+EK.

### 2.3. Standard Hydrolysis of Protein Samples

For the AAAA method, internal standards based on Phe and Tyr homologues were used: FPhe, *M* = 183.18 g/mol (0.0916 g in 20 mL 0.1 N HCl *≙* 25 mM), and HTyr·HBr, *M* = 276.12 g/mol (0.1381 g in 20 mL 0.1 N HCl *≙* 25 mM). From these two stock solutions, a standard solution with 1 mM FPhe and 1 mM HTyr, was prepared. The acidic hydrolysis of the protein samples was performed in a hydrolysis tube: a protein solution with a volume of 20 *µ*L was diluted with 200 *µ*L of 6 M HCl and 5 *µ*L of internal standard was added. Since during this hydrolysis step tryptophan is largely destroyed, only Tyr and Phe were quantified in this work. The thoroughly mixed solution was transferred into a hydrolysis tube. It was evacuated three times and purged with nitrogen. Finally, the tube was evacuated and heated to 107°C for about 22 h in an oil bath. After cooling, the contents were transferred to a 1 mL Eppendorf reaction vessel and evaporated to dryness at 60°C. The pellet was dissolved in 200 *µ*L of high-purity water. For validation purposes, a dilution of 1 : 200 of the NIST BSA standard reference materials was used. The theoretical mass of BSA is 66398.1 g/mol and the numbers of aromatic acids in the sequence are 20 Tyr and 27 Phe. Furthermore, the following complex samples were examined: mouse serum (predilution 1 : 500), human serum (predilution 1 : 500), bovine IgG (stock solution of 0.2 g/L of bovine IgG), and skimmed milk powder (solution of 1 g/L skimmed milk powder). Bovine serum albumin (BSA, 35 *µ*L of a 10 g/L solution) was immobilized on Tresyl-Toyopearl and on Fractogel EMD Epoxy according to the manufacturer's instructions and on two samples of Trisopor Amino 1500, one was immobilized by the glutaraldehyde method and the other by a bis-NHS method based on an activation step with bis-N-succinimidyl diglycolic acid (in dioxane with triethylamine). For the analysis of immobilized protein, each support was dried and 5 mg of the material was hydrolyzed.

### 2.4. Microwave Hydrolysis of Protein Samples

Hydrolysis was carried out in a CEM microwave discovery system with a protein hydrolysis unit. The insert in the reaction vessel had been modified, in which up to five samples in 1.5 mL vials can be hydrolyzed at the same time. This insert had previously been optimized in order to ensure a uniform heat distribution. First, 200 *µ*L of 6 M HCl was added to the vials. Subsequently, 20 *µ*L of protein solution and 5 *µ*L of internal standard solution were added. 10 mL of distilled water was poured into the reaction vessel around the sample vials. A stirring bar in the reaction vessel improves fast temperature equilibration. The method parameters are chosen as follows: standard (measurement of temperature via fiber optics), 50 W maximum power (to minimize bumping), 150°C maximum temperature, 10 min runtime, 20 min hold time, 6.9 bar maximum pressure, and stirring: high. Before the hydrolysis was started, the reaction vessel was evacuated three times and purged with 1.1 bar of nitrogen. In this nitrogen atmosphere, the hydrolysis was performed. After cooling, the samples were transferred to 1.5 mL Eppendorf reaction tubes, evaporated to dryness, taken up in 200 *µ*L of water and analyzed by HPLC. For the examination of the microwave hydrolysis, NIST BSA was used.

### 2.5. Chromatographic Separation of Aromatic Amino Acids

The following separation conditions were used: mobile phase A: water with 0.05% of TFA, mobile phase B: acetonitrile with 0.05% of TFA, flow rate: 0.15 mL/min, column temperature 50°C, and UV detection at 215 nm. 20 *µ*L of sample was injected for analysis. Separation of the aromatic amino acids was performed with a reversed-phase column (Agilent Technologies AdvanceBio Peptide Map 2.1 × 150 mm, 2.7 *µ*m) with a precolumn (Agilent AdvanceBio Peptide Map Fast Guard 2.1 mm × 5 mm, 2.7 *µ*m) on an Agilent 1260 Infinity Bio-Inert Quaternary LC system. A linear gradient from 0% B to 30% B was applied in 40 min. The second step was a cleaning step with a steep gradient from 30% B to 90% B in 2 min and 3 min holding time. After returning to the start condition in 1 min, 19 min of equilibration with mobile phase A was performed.

### 2.6. Data Evaluation

In the case of simple and pure samples, the standard peak integration of the LC system seems to be sufficient. However, due to small peak overlaps with unknown impurities and the lack of selectivity in UV detection, a more sophisticated data evaluation should be considered. To get a more accurate result, a peak fitting was applied to evaluate the peak area (see Supplementary Figure S1 in Supplementary Material available online at http://dx.doi.org/10.1155/2016/7374316). Since the HPLC peaks were not completely symmetric, the asymmetric double sigmoidal function Asym2Sig (Software Origin 9.1G) was found to be most suitable.

### 2.7. Generalized Standard Protocol

Usually, the concentration of the internal standard and of the analyte is chosen to be similar. However, during the hydrolysis process, small matrix peaks might occur. Therefore, a relatively high concentration (25 *µ*M) of the internal standards was considered to be more beneficial. For the sample, approximately 0.1 mM of the respective amino acid would be a suitable concentration. For proteins, approximately 5 *µ*M or 0.3 g/L of BSA would be a perfect sample. A standard protocol could be as follows: 5 *µ*L of internal standard (1 mM) and 20 *µ*L of protein sample (for estimated concentration see above) are added to 200 *µ*L of 6 M HCl. After hydrolysis and evaporation, the sample is dissolved in 200 *µ*L of water. The analysis is carried out on a HPLC system with UV detector (215 nm) and a reversed-phase column. The amino acid concentration is calculated by the ratio of the peak areas of the analyte(s) to internal standard(s) corrected by the extinction factor(s). The protein or peptide concentration is obtained by use of the sequence-derived amino acid content or a mean content of Tyr and Phe in a complex sample obtained by tabled values or by comparison with a reference sample.

## 3. Results and Discussion

### 3.1. Separation of Aromatic Amino Acids on Reversed-Phase Columns

**Figure 2:**
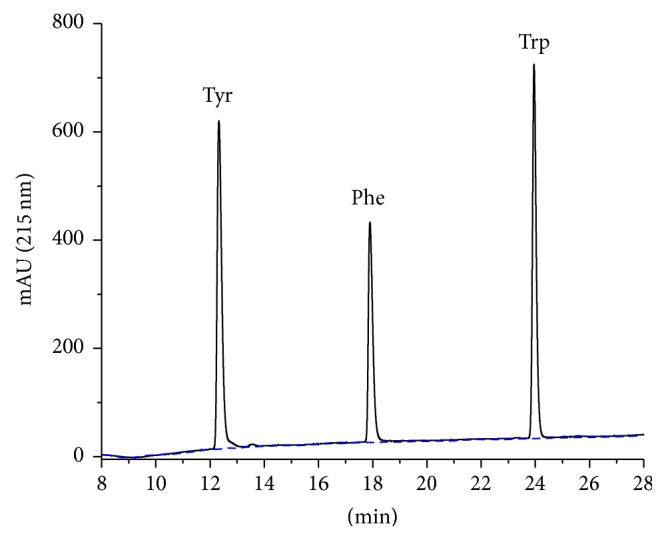
Chromatograms of an amino acid certified reference material (NIST 2389a) diluted in 0.1 M HCl with added Trp (1.25 mM), Gln (2.5 mM), and Asn (2.5 mM). Please note that 17 of these 20 amino acids are not visible in this time window. This is mainly due to weak retention and not due to lack of detectability because most amino acids possess a significant absorbance at 215 nm. In addition, a blank run is shown (dashed).

Figure 2 shows a chromatogram of a standard solution of amino acids (standard reference material NIST 2389a). The small baseline drift around 10 min is most likely caused by the different extinction coefficients of TFA in water and acetonitrile [32]. Obviously, only aromatic amino acids (tyrosine, phenylalanine, and tryptophan) are detected by this setup in the time window between 8 and 28 min (full range between 0 and 40 min is shown in Supplementary Material). The absorbance trace is shown for 215 nm here. At 280 nm only Tyr and Trp can be detected (not shown). Tryptophan was not examined further in this work, due to its instability during acid hydrolysis. Nevertheless, it can be perfectly detected under these separation conditions (Figure 2).

**Figure 3:**
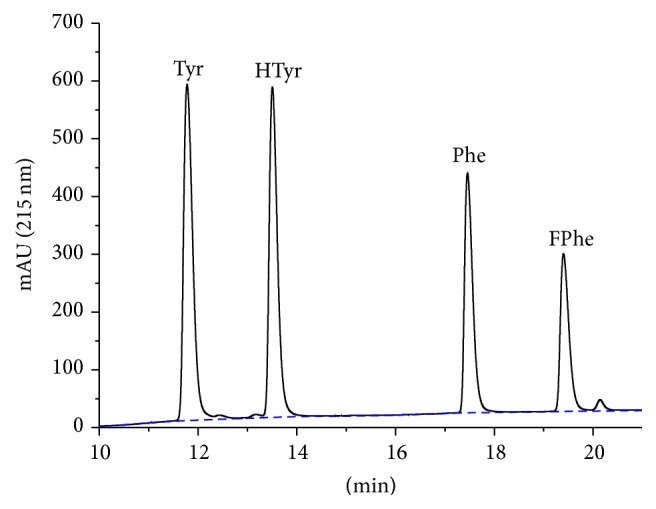
Chromatogram of an amino acid certified reference material (NIST 2389a) in 0.1 M HCl with added internal standards homotyrosine (HTyr) and 4-fluorophenylalanine (FPhe). In addition, a blank run is shown (dashed).

A chromatogram after introduction of two internal standards, homotyrosine (HTyr) and 4-fluorophenylalanine (FPhe), is shown in Figure 3. For UV detection, internal standards are needed, which can be separated chromatographically. However, the compounds should be chosen to be as chemically similar as possible to the respective natural amino acid.

### 3.2. Determination of Tyrosine and Phenylalanine

**Figure 4:**
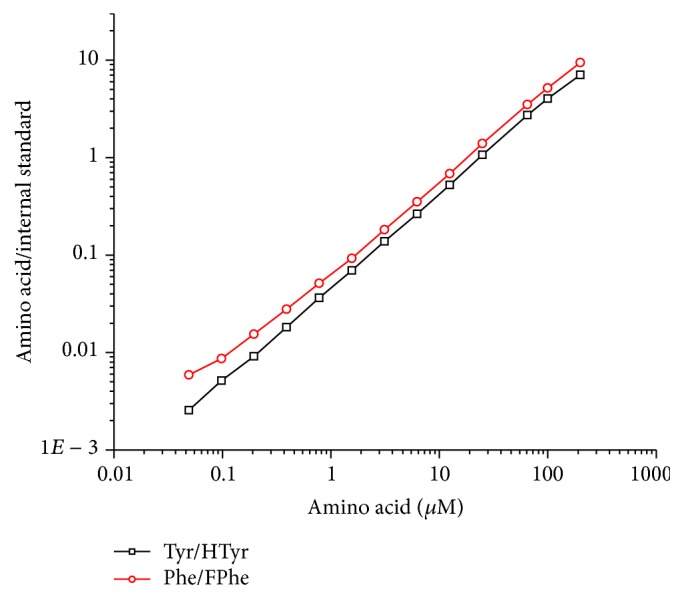
Exploratory calibration lines of phenylalanine and tyrosine (internally calibrated).

For the determination of the concentration of the respective amino acids, the ratio of the peak areas of the amino acid and the corresponding internal standard was used. A proportionality factor is determined, since the extinction coefficients of the internal standard and the amino acid are not identical. The factors were determined to be 0.882 ± 0.027 (*n* = 5) for tyrosine/homotyrosine and 0.673 ± 0.010 (*n* = 5) for phenylalanine/4-fluorophenylalanine. No correction for internal standard purity was applied. Since the HPLC peaks were not completely symmetric and some minor baseline problems occurred with complex samples, an asymmetric double sigmoidal function (Asym2Sig, Origin Software) was used to determine the peak areas throughout (see Supplementary Material). The limit of detection (LOD) estimated from the calibration line in Figure 4 was estimated to be 0.05 *µ*M (~10 *µ*g/L) for tyrosine and phenylalanine (both at 215 nm). The dynamic range of the amino acid determination was examined by extended calibration lines shown in Figure 4. A linear range of more than three decades was obtained, which makes it quite easy to dilute the sample concentration to a valid range.

### 3.3. Protein Determination by Standard Hydrolysis

**Figure 5:**
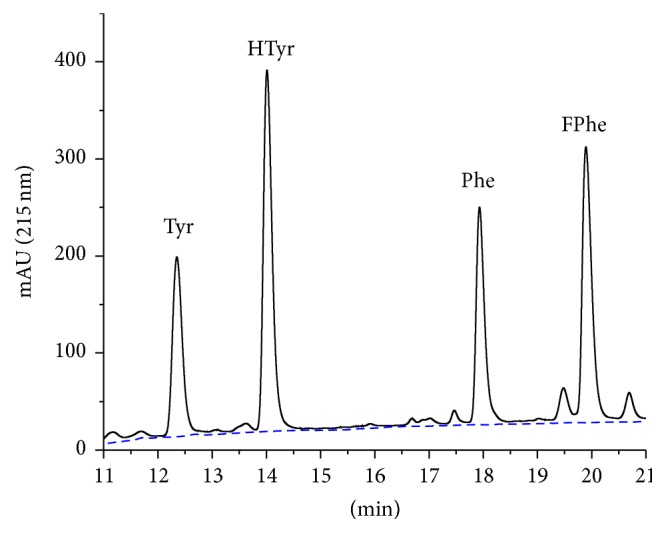
Chromatogram of hydrolyzed bovine serum albumin (NIST SRM 927e, certified reference material) with added homotyrosine (HTyr) and 4-fluorophenylalanine (FPhe) used as internal standards. In addition, a blank line is shown (dashed).

In order to validate the complete method for proteins, a sample of Bovine Serum Albumin (NIST standard reference material SRM 927e, BSA) was analyzed after a conventional hydrolysis in a hydrolysis tube. The chromatogram of the hydrolyzed BSA is shown in Figure 5.

The precision and accuracy of the AAAA method was evaluated considering two criteria, the relative standard deviation (1s) and the recovery of BSA. The certified BSA concentration of the NIST BSA solution based on amino acid analysis is given as 67.38 ± 1.38 g/L. Based on the found amino acid concentrations and the known composition of BSA (20 Tyr and 27 Phe, 66398.1 g/mol), the result of the concentration analysis of BSA was 70.36 ± 3.79 g/L calculated from Phe and 67.87 ± 3.16 g/L calculated from Tyr (*n* = 7). The recovery was 104.4 ± 5.6% based on Phe (215 nm) and 100.7 ± 4.6% based on Tyr (215 nm), respectively. The detection limit of BSA was about 16 mg/L (see Supplementary Material, Figure S2).

### 3.4. Protein Quantification of Complex Samples

**Figure 6:**
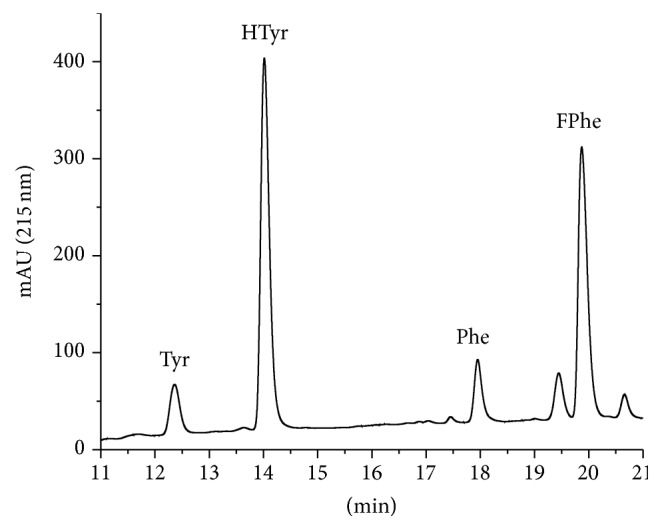
Chromatogram of hydrolyzed mouse blood serum.

**Figure 7 fig7:**
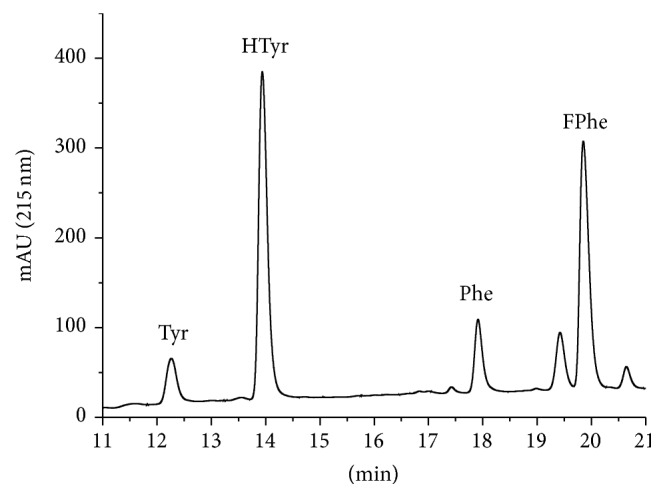
Chromatogram of hydrolyzed human blood serum.

**Figure 8 fig8:**
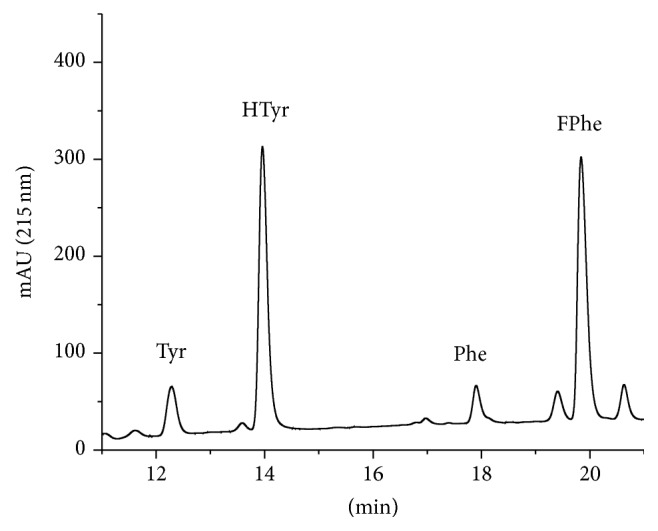
Chromatogram of a sample of hydrolyzed bovine immunoglobulin (IgG).

**Figure 9:**
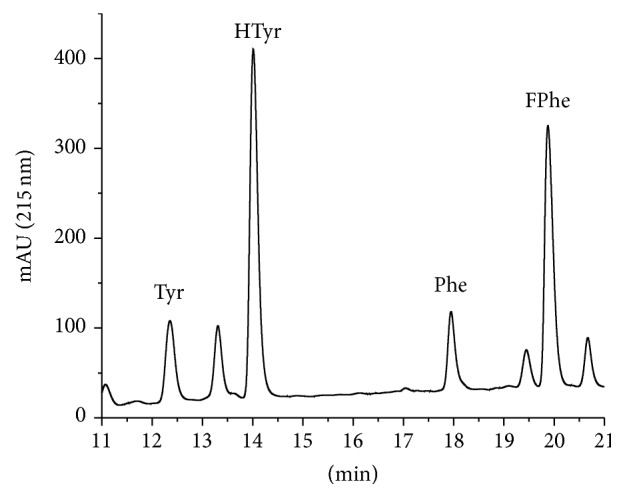
Chromatogram of a sample of hydrolyzed bovine skimmed milk.

This method may also be suitable for the protein determination of complex samples, such as blood serum, plasma, or food samples. Since these samples contain a mixture of many proteins, the concentration cannot be attributed to a protein of known sequence. To use this method quantitatively, a protein (mixture) has to be chosen as a reference to be compared with other samples. This approach assumes that the content of phenylalanine or tyrosine of the reference sample is representative of the other samples. As examples, sera from mouse (Figure 6) and human (Figure 7) origin were hydrolyzed and analyzed to examine the ability to obtain baseline-separated peaks under these conditions. In addition, bovine IgG (Figure 8) and bovine skimmed milk (Figure 9) were investigated. For all samples no obvious interference peaks could be found in the respective detection window.

### 3.5. Discussion

The results presented in this paper show the potential of this method for the quantification of aromatic amino acids and the quantification of pure proteins and crude protein preparations, including covalently immobilized on solid supports (see Supplementary Material). In Figure 2 it could be shown that tyrosine, phenylalanine, and tryptophan can be perfectly separated on a standard HPLC system with UV detector, without any derivatization. Since tryptophan is not stable under acidic hydrolysis conditions used to cleave peptides and proteins, its quantification has not been examined in more detail here. However, the excellent retention of tryptophan is an indication that even other (rare) aromatic amino acids might be determined with this separation protocol. The calibration can be performed by different approaches, such as conventional external calibration or internal calibration by isotope dilution, if a mass spectrometric detection is possible. However, we decided to use calibration by internal standards, which can be chromatographically separated. For this purpose homotyrosine (HTyr) and 4-fluorophenylalanine (FPhe) were chosen. Both are readily available and show a similar retention shift to the natural amino acid (Figure 3). The compounds are not analytically pure, which leads to some additional minor peaks. This, has no major impact on quantification, if this purity issue is corrected with the peak ratio, which is anyway necessary due to the different extinction coefficients of internal standard and analyte. This approach reduces the instrumental requirements to a standard HPLC system with a simple UV detector, which should be available even in less equipped laboratories. The dynamic range of the method is more than satisfactory; about four decades could be reached for both amino acids. A limit of detection (LOD) of 0.05 *µ*M for Phe and Tyr could be shown based on an injection volume of 20 *µ*L. These LODs are equivalent to 1 pmol absolute or ~150 pg. The limit of detection for proteins is more difficult to determine, since background problems caused by the hydrolysis step might be more prominent. Based on the NIST SRM 927e reference material (BSA), a LOD of <16 mg/L (~200 nM) was estimated. In absolute amounts, 4.5 pmol or 300 ng BSA could be detected (injected amount).

In Figure 5 a chromatogram of hydrolyzed bovine serum albumin (BSA) is shown. From these data alone, it cannot be decided whether the proposed method leads to valid results. Since the sample was a reference material with known BSA and therefore Tyr and Phe content, the found amount can be directly compared with the certified value. The excellent agreement of the concentrations obtained by Tyr and Phe and their very good agreement allows the assumption that the proposed method is able to quantify proteins with very good accuracy and precision (~5%).

Figures 6 [Fig fig7] [Fig fig8]–9 show the chromatograms of hydrolyzed real samples of complex composition. It is surprising that without any sample clean-up or other selective steps only very few additional peaks can be observed under the conditions chosen. Hence, not only pure single proteins or purified protein mixtures can be characterized by AAAA, but also quite crude samples, such as milk. Since the determination of the protein content of complex samples is highly method dependent, we do not give any “reference values” here.

Finally, the application of AAAA to covalently bound proteins was tested on solid supports, for example, porous glass or beads made of synthetic polymers (see Supplementary Material). This analytical question is particularly challenging, since most traditional methods fail under these conditions. In Figure S3 some exemplary chromatograms are shown, which reflect the efficiency of the respective immobilization methods. However, additional validation work is necessary in this case to establish AAAA as a routine method for the quality control of protein immobilization.

## 4. Conclusions

In this paper, we could show that the use of aromatic amino acid analysis (AAAA), mainly based on the determination of tyrosine and phenylalanine on a standard HPLC system with a UV detector, is an alternative to other methods of protein quantification, which either are more complicated, expensive, and time-consuming or are much less accurate, prone to matrix effects or fraudulent manipulation (e.g., addition of melamine) of the samples. The chromatographic determination of aromatic amino acids in combination with fast microwave hydrolysis could be a reasonable compromise between time and effort on the one hand and analytical reliability and accuracy on the other hand. The limits of detection regarding proteins are comparable to colorimetric assays, however offering better accuracy, if any information about the amino acid composition is available. Even without such information, the protein determination should be better than colorimetric methods calibrated with the wrong protein, which is the rule in practice and not the exception. In addition, it could be shown that reproducibility of AAAA is very good. A routine application of AAAA would significantly improve the quality of many protein determinations, which are performed with simple and cheap, but less accurate methods. In addition, quality control (QC) protocols of biochemical products based on peptides and proteins, irrespective whether being pure compounds, such as pharmaceutical drugs, or consisting of complex protein mixtures or extracts, such as milk powder, might be established based on AAAA. The lack of quantitative and convenient methods to determine covalently bound proteins on solid supports and surfaces might be overcome with the method shown here. The instrumental requirements are very moderate and hence this approach could be successfully established even in less specialized labs and environments. Due to the simplicity and low cost, AAAA might be established for applications, where amino acid analysis was not even considered.

## Supplementary Material

The supplementary material shows an extended time window for chromatograms, details of the data evaluation (peak fitting), the estimation of the limit of detection (LOD) of proteins, immobilization protocols of bovine serum albumin (BSA) on solid supports and the quantification of covalently immobilized protein.
