# Supplementary material for: Protein Quantification by Derivatization-Free High-Performance Liquid Chromatography of Aromatic Amino Acids
Source: J Amino Acids. 2016 Jul 31;2016:7374316. doi: 10.1155/2016/7374316 (PMC4983383; doi:10.1155/2016/7374316)
Supplement: Supplementary file 1 — The supplementary material shows an extended time window for chromatograms, details of the data evaluation (peak fitting), the estimation of the limit of detection (LOD) of proteins, immobilization protocols of bovine serum albumin (BSA) on solid supports and the quantification of covalently immobilized protein. [file 7374316.f1.pdf]

Supplementary Material

Protein quantification by derivatization-free high-performance liquid chromatography of aromatic amino acids

Almut Hesse and Michael G. Weller*

Bundesanstalt für Materialforschung und -prüfung (BAM)
Division 1.5 Protein Analysis
Richard-Willstätter-Strasse 11
12489 Berlin, Germany
* Correspondence: michael.weller@bam.de
Tel.: + 49-30-8104-1150, Fax. + 49-30-8104-71150

1. ***Extended time window for chromatograms***
2. ***Details of the data evaluation (peak fitting)***
3. ***Estimation of the limit of detection (LOD) of proteins***
4. ***Immobilization protocols of bovine serum albumin (BSA) on solid supports***
5. ***Quantification of covalently immobilized protein***

1. Extended time window for chromatograms

For illustrative purposes, chromatograms over the extended time window between 0-40 min are given below. Figure 2a is an example of a standard solution of 20 amino acids, and Figure 7a shows a hydrolyzed real sample (human serum).

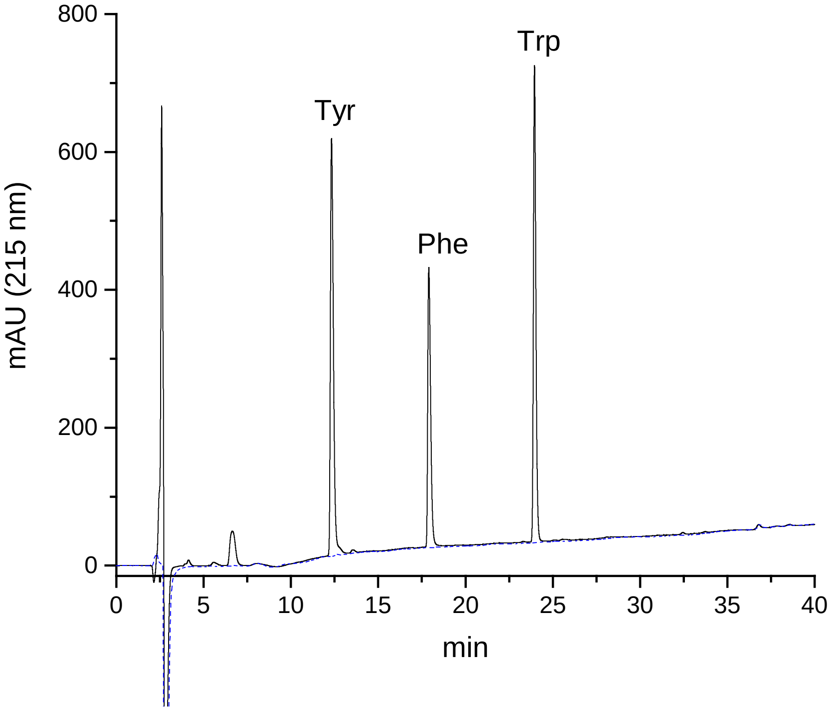

Figure 2a (Extended time range of Fig. 2): Chromatograms of an amino acid certified reference material (NIST 2389a) diluted in 0.1 M HCl with added Trp (1.25 mM), Gln (2.5 mM) and Asn (2.5 mM).

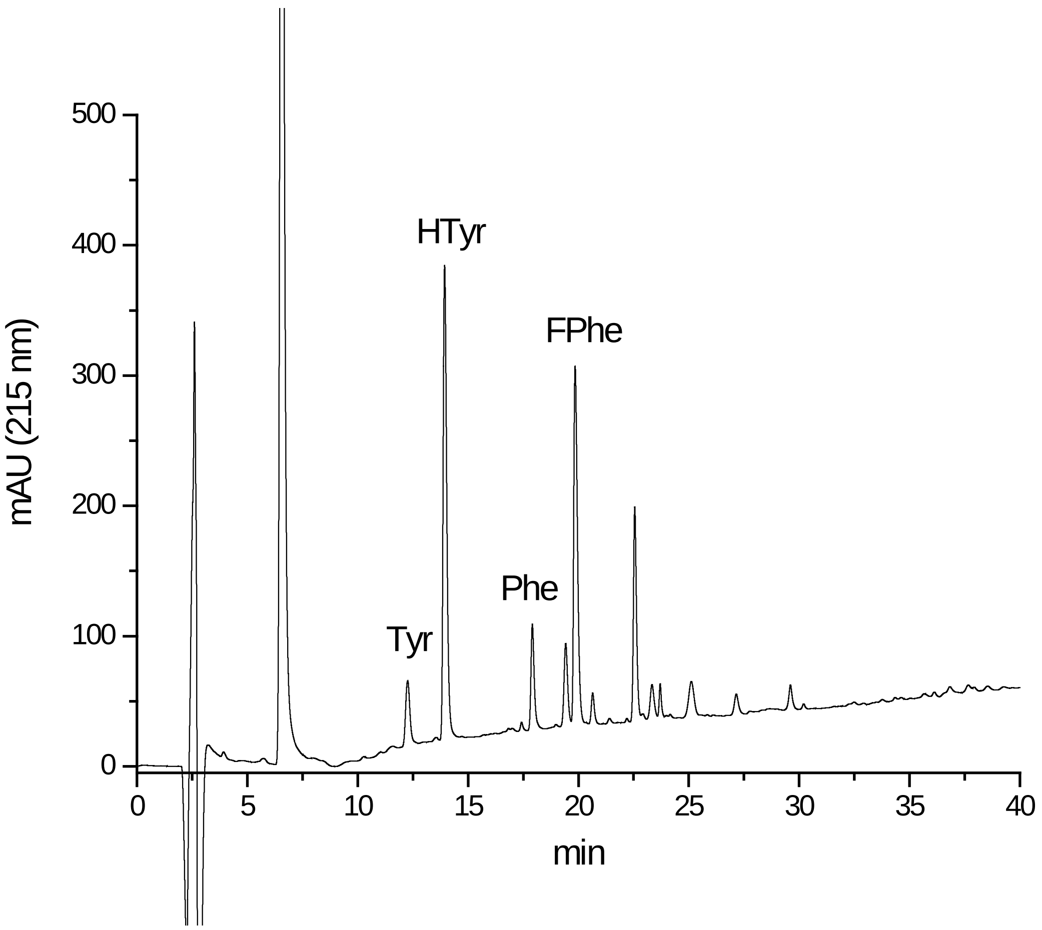

Figure 7a (Extended time range of Fig. 7): Chromatogram of hydrolyzed human blood serum.

2. Details of the data evaluation (peak fitting)

Some amino acid peaks displayed small shoulders or other partially overlapping peaks. With standard HPLC software, the integration of such peaks might be difficult. Therefore, we used a special function of the software Origin to fit all peaks and to reduce inaccuracies based on integration problems (Example Fig. S1). The standard function Asym2Sig (Origin) is defined as:

$$y = y_0 + A \cdot \frac{1}{1 + e^{-\frac{x - x_c + w_1/2}{w_2}}} \cdot \left(1 - \frac{1}{1 + e^{-\frac{x - x_c - w_1/2}{w_3}}}\right)$$

Here y_0 describes the offset, A is the amplitude, x_c is the center of the peak, w_1 is the full width of the half maximum, w_2 and w_3 are the variances of both sides. This approach also facilitates the establishment of a robust baseline.

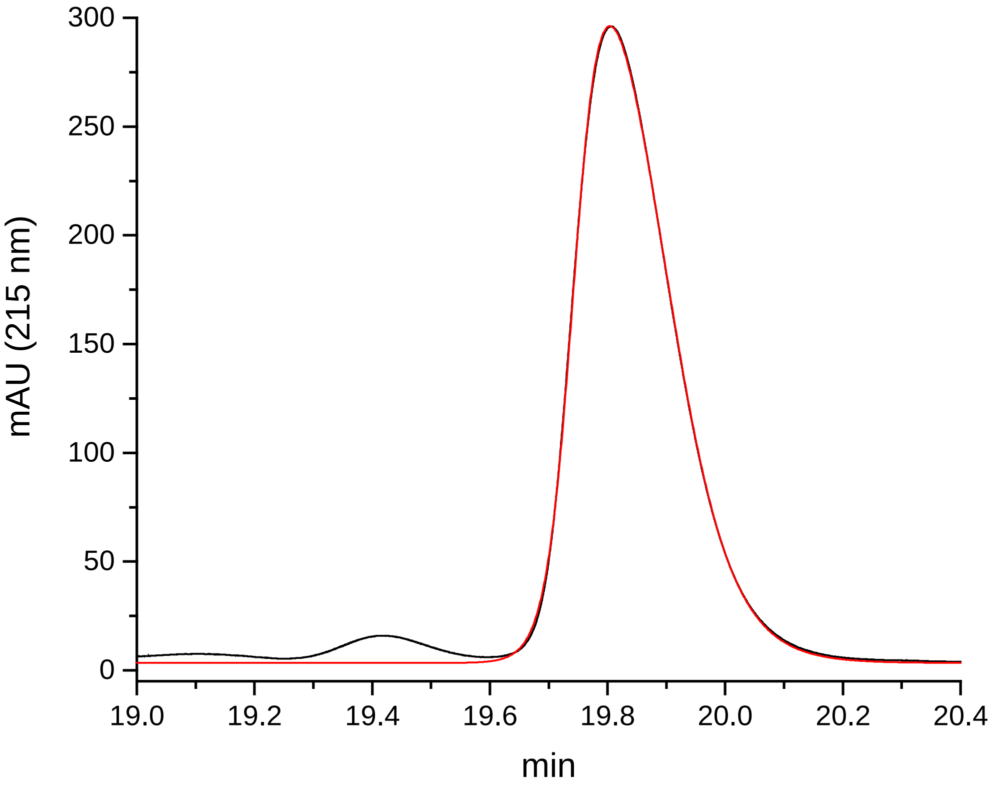

Figure S1: The 4-fluorophenylalanine peak of hydrolyzed serum albumin (NIST 927e, certified reference material plus internal standards) in black and the fitted peak with the Asym2Sig model (red).

3. Estimation of the limit of detection (LOD) of proteins

A certified reference material of bovine serum albumin (BSA) was used to estimate the detection limit of proteins (Fig. S2). The calculation is based on the phenylalanine peak at about 17.1 minutes. The concentration of 16 mg/L showed a significant increase of the peak area. The signals detected with lower concentrations are suspected to be caused mainly by a carryover during the microwave step. Hence, some improvements seem to be possible by optimization of the hydrolysis step.

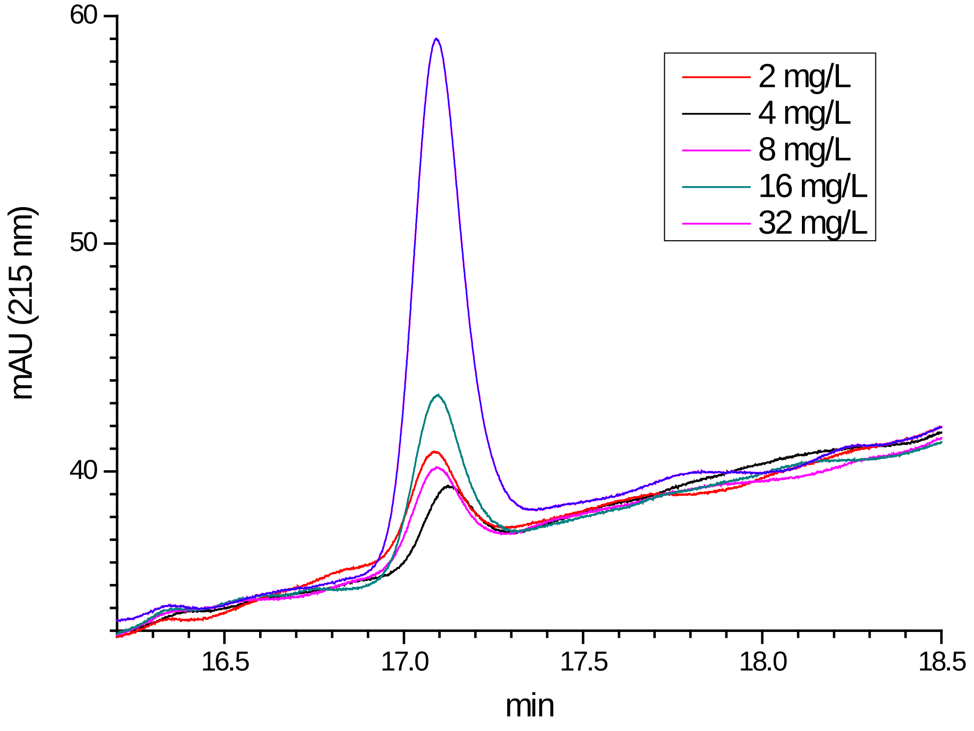

Figure S2: Phenylalanine peak from the chromatogram of hydrolyzed serum albumin (BSA) solution (NIST SRM 927e, certified reference material), injection of 20 µL. The limit of detection (LOD) for BSA was estimated to be below 16 mg/L.

4. *Immobilization protocols of bovine serum albumin (BSA) on solid supports*

30 mg of solid support were used for immobilization. To activate the Amino Trisopor 200 µL of a solution with 12.5% glutaraldehyde (Sigma-Aldrich, G7651) and 15 mg sodium cyanoborohydride (Acros Organics, New Jersey) were added to the support. After washing five times with PBS, 35 µL of 10 mg/mL BSA solution (Sigma-Aldrich 98%) with additional 15 mg sodium cyanoborohydride was used for the immobilization and incubated for 15 h. Subsequently, the support was washed with PBS, blocked with 200 µL 0.1 M Tris and 15 mg sodium cyanoborohydride and finally the support was washed three times with PBS.

An alternative activation method was performed by mixing 38.5 mg bis-N-succinimidyl glycolic acid and 10 µL triethylamine (Sigma-Aldrich, BioUltra) dissolved in 600 µL of anhydrous dioxane (Sigma-Aldrich 99,8%) and subsequent incubation of the aminated solid support for 3 hours. After washing four times with dioxane and once with PBS, 35 µL of 10 g/L BSA solution was added and incubated for 15 hours. Finally, the support was washed with PBS.

To immobilize proteins on the supports Fractogel EMD Epoxy and Tresyl Toyopearl, the protein solution (20 g/L BSA) was diluted 1:1 with 0.1 M carbonate buffer (pH 9.6) with 0.15 M NaCl, for Fractogel EMD Epoxy and 0.1 M sodium hydrogen carbonate (pH 8) with 0.5 M NaCl, for the Tresyl Toyopearl support. 35 µL of this protein solution was incubated for 42 h (Epoxy) and for 4 h (Tresyl). Subsequently, the support was washed with PBS, blocked with 200 µL of 0.1 M Tris and finally the support was washed three times with PBS.

After drying, 5 mg of each support and 20 µL of internal standard solution was diluted in 200 µL of 6 M HCl, hydrolyzed and examined by AAAA to determine the immobilization yield.

5. Quantification of covalently immobilized protein

In order to quantify proteins, which have been immobilized on polymeric or inorganic supports, such as glass or quartz, quite a few methods have been proposed. Most of them have to be considered to be semi-quantitative at best, since the surface or the pore structure might heavily interfere with the quantitative determination. We tested BSA immobilized (Method see Supplementary Material) on support materials based on porous glass and polymeric beads (see Fig. S3). The materials were subjected to the usual hydrolysis procedure and finally analyzed chromatographically. After hydrolysis of the supports the immobilization rate was determined by AAAA. Both, tyrosine and phenylalanine could be determined without difficulty. The protein capacity was calculated by AAAA to 5.7 mg/g by glutaraldehyde immobilization, 4.1 mg/g by bis-NHS ester and 2.2 mg/g for the Tresyl Toyopearl resin. Unfortunately, materials with active epoxy groups lead to unusual low values, which might be caused by insufficient blocking. In addition, materials based on carbohydrates (e.g. Sephadex) are not suitable for this standard protocol, since they decompose to dark solutions during the acidic hydrolysis. This method is promising to specify the immobilization yield of affinity material based on synthetic polymers or porous glass supports. A validation of this solid phase method is not available, yet, and hence, these results should be seen as preliminary, merely to illustrate the versatility of the method.

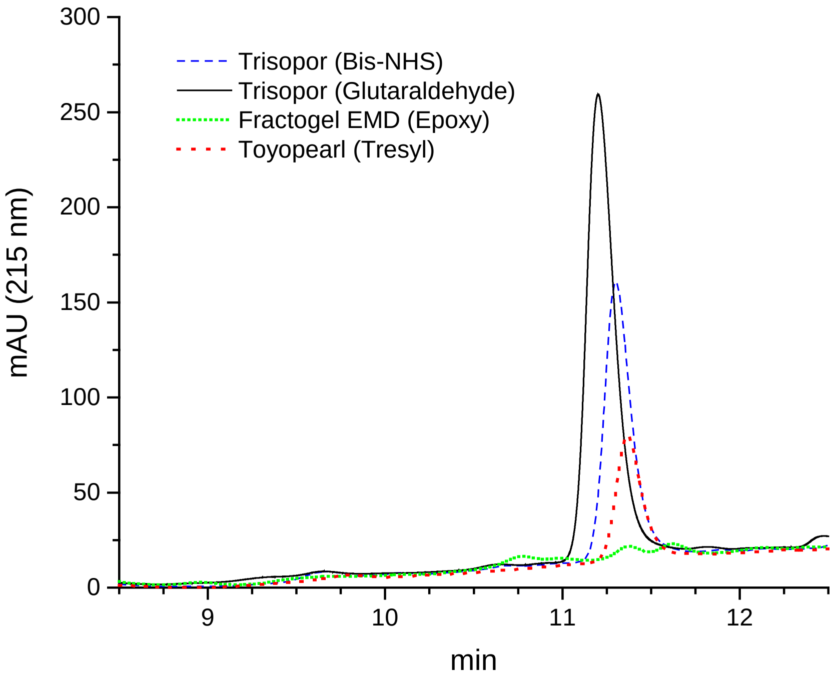

Figure S3: Tyrosine peaks of bovine serum albumin (BSA) immobilized on different solid supports: Aminated porous glass, activated with bis-N-succinimidyl diglycolic acid; aminated porous glass, activated with glutaraldehyde; Tresyl-activated Toyopearl and epoxy-activated Fractogel EMD obtained by AAAA.
